# Gene-Based Clustering Algorithms: Comparison Between Denclue, Fuzzy-C, and BIRCH

**DOI:** 10.1177/1177932220909851

**Published:** 2020-04-01

**Authors:** Martin C Nwadiugwu

**Affiliations:** Department of Biomedical Informatics, University of Nebraska Omaha, Omaha, NE, USA

**Keywords:** Gene-based clustering, Denclue, Fuzzy-C, BIRCH

## Abstract

The current study seeks to compare 3 clustering algorithms that can be used in gene-based bioinformatics research to understand disease networks, protein-protein interaction networks, and gene expression data. Denclue, Fuzzy-C, and Balanced Iterative and Clustering using Hierarchies (BIRCH) were the 3 gene-based clustering algorithms selected. These algorithms were explored in relation to the subfield of bioinformatics that analyzes omics data, which include but are not limited to genomics, proteomics, metagenomics, transcriptomics, and metabolomics data. The objective was to compare the efficacy of the 3 algorithms and determine their strength and drawbacks. Result of the review showed that unlike Denclue and Fuzzy-C which are more efficient in handling noisy data, BIRCH can handle data set with outliers and have a better time complexity.

## Introduction

Clustering is a useful method that groups items based on certain similarity measures for understanding the structures, functions, regulation of genes, and cellular processes obtained from gene expression data and providing more insight on a given data set.^1,2^ It is an essential step in analyzing biological data (eg, omics data) to deduce unknown functionalities of the units of data.^3^ The purpose of using clustering methods is to group together objects more similar to one another, which is quite useful in bioinformatics where it is implemented to identify tumors from patients and molecular subtypes of disease.^4^ However, for every clustering problem, there exists an appropriate algorithm.^5^ Gene-based clustering regards the genes as objects and samples as features; the technique helps to identify homology by separating genes in clusters and allowing a noticeable difference among them which is vital in finding patterns for designing vaccines, classifying genes according to their related functions, and analyzing diseases.^1^ Patterns for designing vaccines are obtained by computational approaches studying proteome of bacteria and identifying those that have catastrophic roles in cells,^6^ while clusters of *protein*-*protein interactions* help in analyzing diseases, because similar diseases are caused by proteins with similar functions.^7^

**Figure 1.**
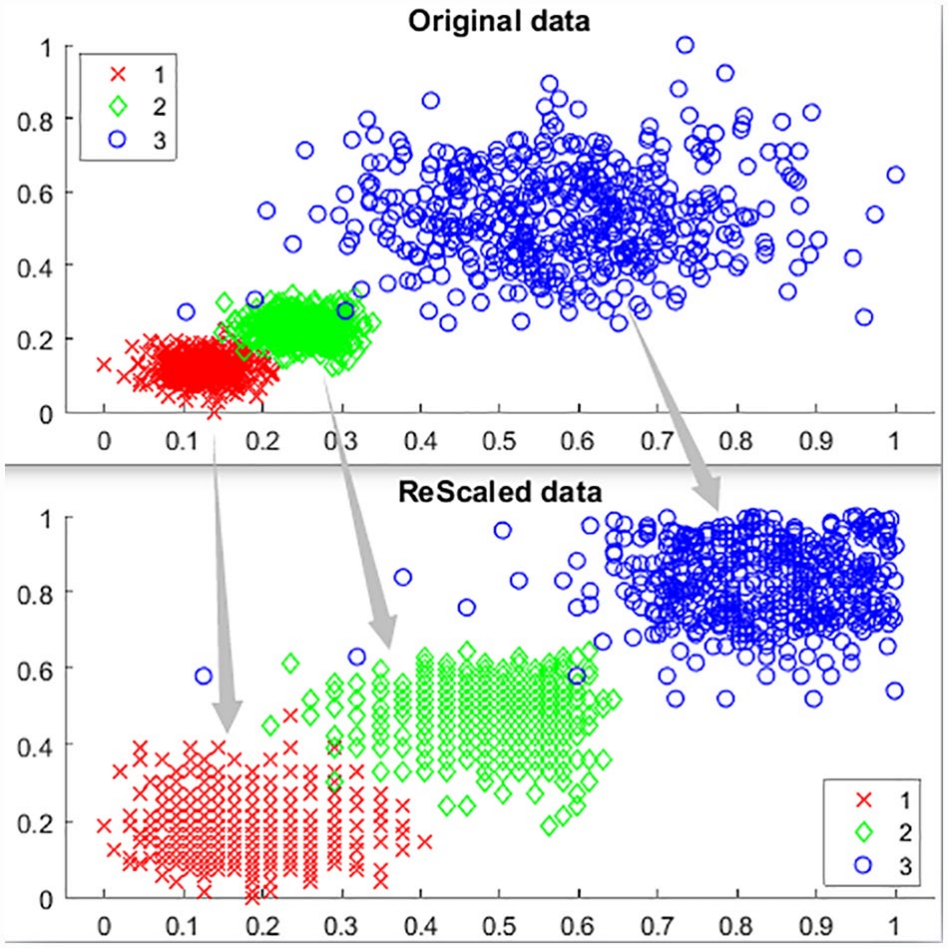
Clustering algorithm: Example of a clustering algorithm where an original data set is being clustered with varying densities.^10^

Identifying genes with similar characteristics, for example, in gene expression data via cluster analysis is an important focus in bioinformatics research.^8^ Clustering helps identify genes with patterns of similar expression in gene expression data analysis, because it group genes that are more similar to each other, so that genes with similar functions or pattern of variations can be found. Three gene-based clustering algorithms (Denclue, Fuzzy-C, and Balanced Iterative and Clustering using Hierarchies [BIRCH]) were selected representing 3 traditional clustering techniques: density-based, soft-clustering, and hierarchical clustering approaches, respectively. Computational intelligence clustering methods using self-organizing maps are now increasingly being used in bioinformatics due to the limitations of traditional clustering techniques.^9^ These methods incorporate artificial neural networks and competitive learning, and have been implemented in unsupervised clustering of metabolites and transcriptome profiles.^9^ An example of the results of a clustering method using the density-based approach is shown below in Figure 1.

The density-based clustering approach identifies clusters of co-expressed genes in a multidimensional data set separated by high-dense and sparsely dense areas. This method could be computationally inefficient depending on input parameter as it identifies outliers and internally embedded clusters which increase noise within the data set.^11^ The soft-clustering approach, on the contrary, has sample points in the cluster which have membership function that indicates whether they have strong or weak association to a given cluster; while the hierarchical approach build a series of nested clusters with distinct characteristics represented as dendrogram, showing similarity between the clusters and formation of clusters.^1^

Clustering algorithms have been used in modeling drug focus by studying gene expression data to isolate clusters that are implicated in pathogenic attacks, differential expression of genes related to inflammatory mechanisms.^1^ For example, hierarchical clustering has been used in profiling the mycobacterium tuberculosis in HIV/AIDS research to determine genetic markers and genes for targeted treatment, and to distinguish between asthma and normal cells from genome-wide transcriptional patterns.^1^ Although the study focuses on 3 selected algorithms, there exists a range of other clustering algorithm that has proven to be beneficial in bioinformatics research; an example is the k-means algorithm that was used in the discovery of subtypes of parkinsonism, and in detecting stages of breast cancer malignancy on mammogram based on the size of cancer.^12,13^ Much can be learned by comprehensively comparing clustering methods and how they could be implemented in many possible scenarios.^1^ The aim of the study was to compare clustering algorithms used in gene-based clustering analysis, their clustering procedure, their efficiency, and their capability in handling noisy, big dynamic data, and extracting true clusters out of it.

The remaining sections of this article will highlight the purpose of the study and provide a brief overview of the algorithms, including a pseudocode of how they are implemented. The study will try to answer the research question by formally implementing an example of how the algorithm works using python and displaying the results. Next, a comparison between the clustering algorithms will be highlighted on a table. The article will conclude with a brief discussion on the topic, the limitations, and lesson learned.

## Purpose of the Study

The purpose of the study was to compare clustering algorithms used in gene-based clustering analysis, their clustering procedure, and how they are implemented in the extraction of true clusters from recent literature. The rationale for comparing the algorithms is that there exist several clustering algorithms that produce different optimal result depending on some criteria such as sample size used. Therefore, it is vital to compare the efficacy of clustering algorithms to provide preliminary information for researchers choosing to adopt a more suitable algorithm.^14^ Python programming language was used to test and evaluate the implementation of the 3 clustering algorithms for efficiency; thereafter, manual visual inspection was used to validate the clusters. The study objective to guide the methodology and analysis was to try to answer the question: *Which of the 3 clustering algorithms was more efficient and best extracted true clusters*?

### Overview of the selected algorithms

#### Denclue algorithm

Denclue is a density-based clustering algorithm that identifies clusters of dense areas and nondense areas.^15^ It is simply clustering based on density that starts by creating a network of portions of the data set, and using the influence function, which are points going to same local maximum describing the outcome of data points within the same clusters, to calculate the density function.^16^ It uses a generic form that combines hierarchical and partitioning clustering methods.^11^ Denclue is a good algorithm for data sets with a lot of noise because it allows for centralized description of irregularly shaped clusters in a data set with high dimension by identifying outliers as data points with low cardinality and excluding them so that only relevant data points are clustered.^1^ Clusters are determined using hill climbing by identifying density attractors (highest value of density function), and data points of the density attractors as belonging to the same cluster; so calculating the density attractors or local maxima is important for determining the clusters.^1^

Denclue, when implemented in gene-based clustering, can show dense and nondense areas of genes that correlate to complexes and patterns of gene associations. When implemented with a simulated data of a pliable peptide, it shows better efficacy than DBSCAN which is another type of density-based clustering algorithm.^17^ Denclue follows the pseudocode and algorithm below as suggested by Kumar and Batra.^11^

**Problem:** To determine density attractors and associated data objects using hill climbing, and merging the initial clusters if possible.

**Input:** x, y (location of the object)

**Output:** $e^{-d{\left(x,y\right)}^{2}}/2\sigma^{2}$ (density attractors) (1)

**Variable definition** :

x and y: influence functions

d(x, y): euclidean distance

ƒGauss(x, y): gradient

(2) $$fgauss\left(x,y\right)=e^{-d{\left(x,y\right)}^{2}}/2\sigma^{2}$$

The above equation shows the gradient of 2 genes x and y (influence function), where the Euclidean distance is d(x, y), and σ is the radius of the neighborhood containing x gene. The σ tells how swiftly the effect of changes of y on x decreases as the distant point between y and x increases.^16^ The influence of the entire data points x_1_ ε X on another point y_1_ ε Y is measured by summing the density function on (y_1_). This technique (density estimation) uses influence functions measure of a point x in relation to another point y, and the effect of changes of x on the density of point y decreases as the distant point between them (x, y) increases.^16^ The algorithm works by constructing a map for the database of genes (eg, in a biological scenario) and determining the populated genes. Next, it connects the populated gene nodes to construct a map. The time complexity is *O* (*NlogN*), and it uses hill climbing method (clustering data points of the local maxima) to find the density attractors of the same path which are connected to form the final clusters.

### Limitation and future direction of the density-based clustering approach

Implementation becomes complicated when data set becomes quite large or when the right parameters are not selected, and the data are high dimensional and not uniform.^1,17^ A modified form of the algorithms that effectively work with large data set would be an improvement to this clustering approach. The modified form would handle nonuniformity by calculating the mean of the populated data sets, the connection between each populated data set and other data sets by the distance between their mean. Thereafter, the highly connected data sets having the same path would form the clusters with assigned values.^17^ This could be a way of handling large, nonuniform data sets.

#### Fuzzy-C algorithm

**Figure 2.**
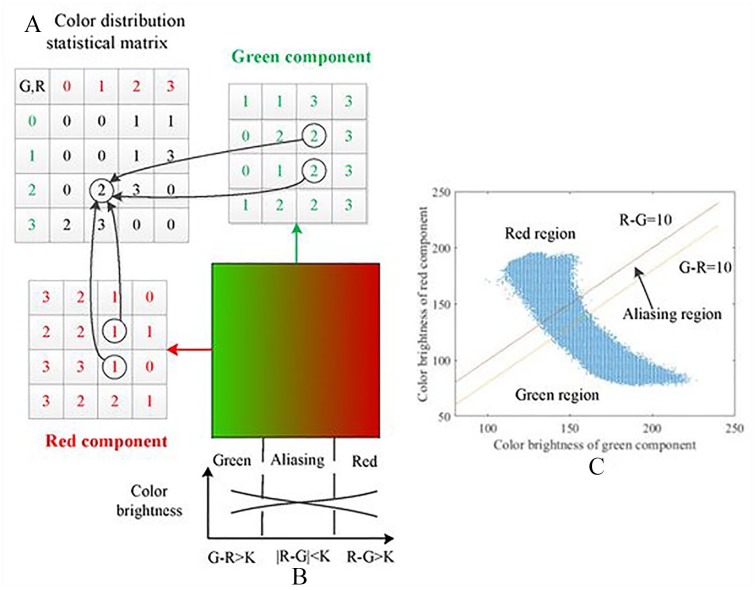
Fuzzy-C algorithm: Example of an image data being clustered with Fuzzy-C with (A) showing determination of degree of membership, (B) showing the image data, and (C) showing the output of the clustering.^19^

Fuzzy-C was introduced in 1981 by Jim Bezdek; the algorithm typically groups data into clusters and obtains membership degree of data points to each clusters.^18^ It is a soft-computing algorithmic approach that typically states that for a single data point X that belongs to different clusters *C*_1_, *C*_2_*,. . .C*_n_, the values of the data points for each clusters will be calculated to determine its degree of association/membership, and this value will be updated on each iteration.^11^ The algorithm implementation as shown in Figure 2 minimizes the criterion for association, with respect to the degree of membership value Uij, and the distance dij (distance between the objects and the corresponding cluster).^20^ Although Fuzzy-C has difficulties with cluster validity and inability to deal with outliers, it is still a clustering method used for microarrays, a dated technique still important in microbiome research, simple visualization, and to validate results from modern sequencing techniques; Fuzzy-C also have the advantage of being able to converge, ie, the addition of sample points across all clusters is zero, and to cluster overlapping sample points.^1^ Future improvement of the algorithm should consider resolving issues related to cluster outliers.

Fuzzy-C is implemented and tested in gene clustering to show how the algorithm connects each gene to clusters, where the gene is a real member using soft boundaries, ie, assigning data point values that represent close association to clusters, thereby allowing them to be members of more than one clusters. If the gene is a member of a cluster, it is given a value of 1, and a value of 0 where it has weak association.^21^ The algorithm works by specifying the number of clusters (k) and randomly assigning data point’s coefficients for the clusters. This step is repeated until the iteration is complete or the sensitivity threshold (changes between 2 iterations) is no longer possible. The cluster centroid and its coefficients of being in the clusters are then computed. The algorithmic pseudocode and formula can be summarized below.

**Problem:** Given a data set find the degree of membership of x in all clusters.

**Input:** Uij (degree of membership of x in cluster j)

**Output:** Uij + 1(update of membership and cluster center, until Uij + 1 –

Uij<0<1)

(3) $$\sum_{i=1}^{n}\sum_{j=1}^{k}u_{\mathrm{ij}}^{m}\;d({\vec{X}}_{i},\;{\vec{C}}_{j})\;,\;1<\;m\;<\;\;\infty$$

**Variable definition:**

Data set X = x1, x2 {x1, x2,. . .xn} ⊆=R^nxq, n is the number of samples, j is the cluster going from 1 to k, Cj is the centre of the cluster, q is the dimension of the sample xj (j= 1,2,. . ., N). The formula can be seen above.

The time complexity for Fuzzy-C algorithm is *Near O(N*). In essence, for a set of gene cluster having isolated data points, the Fuzzy-C algorithm can create soft boundaries and assign the data point to a cluster based on its strong membership.

## Balanced Iterative and Clustering using Hierarchies

BIRCH was developed in 1996 by a group of researchers in Wisconsin. It is an incremental and dynamic clustering algorithm that follows a hierarchical clustering technique for databases by incrementally constructing a clustering feature (CF) tree, which is a subcluster of data points or better described as a tree-like representation of data points in a data set.^22^ Best clustering is achieved by multi-scanning, and having more available memory which maximizes good result.^11^ BIRCH is an incremental clustering algorithm that has 4 phases. The first phase scans the entire data set and constructs a first-in memory CF tree. The second (constructs smaller CF tree) and fourth (cluster filtering) phases are optional, whereas the third phase applies agglomerative hierarchical clustering algorithm to the subclusters.^23^

**Figure 3.**
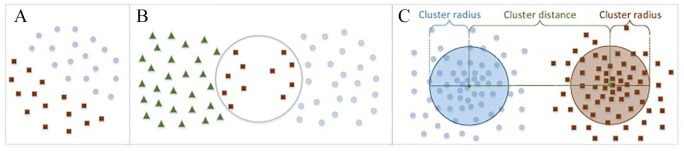
BIRCH: A data set showing (A) group of combined clusters, (B) cluster radius and distance, and (C) categories of different clusters with each containing similar elements.^24^

The advantage of BIRCH is that while other algorithms have trouble dealing with outliers and large data sets, it infers the best obtainable subclusters while limiting input/output and has the capacity to slowly but progressively group multidimensional metric to produce clusters of the best quality. The algorithm works by scanning a database to build a CF tree in-memory—a multiphase clustering to maintain the inherent structure of the data. It then clusters the nodes of the CF tree using an arbitrary clustering algorithm. The time complexity is *O(N).* An example of the method is highlighted in Figure 3.

**Problem** : clustering data points from N-dimensional data

**Input:** N-dimensional data points x_1_, x_2_, . . .X_n_.

**Output:** CF = (N, LS, SS)

**Variable definition:**

CF = (N, LS, SS) and

N – Number of data points of a particular cluster

LS – Linear sum of points N

SS – Squares of the points N

(4) $$\begin{array}{l} \mathrm{where}\;\;\vec{\mathrm{LS}\;}=\overset{N}{\underset{i=1}{\;\Sigma }}\vec{X_{i}}\;\;\mathrm{is}\;\mathrm{the}\;\mathrm{linear}\;\mathrm{sum}\;\mathrm{and}\;\\ \;\;\;\;\;\;\;\;\;\vec{\mathrm{SS}}=\;\sum_{i=1}^{N}(\vec{X_{i}}{)}^{2} \end{array}$$

**Example:** If we have 2 cluster with 5 number of data points within each cluster, (3,4; 2,6; 4,5; 4,7; 3,8) and (6,2; 7,2; 7,4; 8,4; 8,5) the cluster frequency can be calculated as:

L = 5

LS = (16, 30) for cluster 1 and (36, 17) for cluster 2

SS = (54, 190) for cluster 1 and (262, 61)

CF = (10, (52, 47), (316, 251))

### Limitation and future direction of the approach

Due to the limited number of data points a CF tree can hold, it may not give real-life simulation of natural clusters. Not only that, because it uses radius and diameter in cluster associations, it may not execute properly if the clusters are not spherical in nature. Future improvement to the algorithm would have to incorporate these drawbacks.

## Implementing the Algorithms

Objective: To create clusters that show implementation of the 3 different algorithms, and using manual visual inspection to validate if it followed the definitions and best extracted true clusters?

The clustering algorithms were implemented using Python programming accessed from PyCharm Community Edition 2.4 on Windows 10 Education Operating System edition with an x64-based processor and installed memory of 4 gigabytes. The sklearn clustering suite which has about 13 different clustering classes was used to generate data with clusters. These data were used to show how the algorithm would work. The implementation of the algorithms was adapted from GitHub example, modified, and archived in the GitHub repository.^25,26^

## Result

**Table 1.** Comparison between Denclue, Fuzzy-C, and BIRCH.^11,27^

Algorithms	Key idea	Limitation	Complexity	Cluster Shape
Denclue	It utilizes the influence points between data points of network to represent the density function and is capable of handling high dimensional data	Hill climbing—may not move toward one point maximally	*O*(*NlogN*) (time)	Arbitrary
Fuzzy-C	Minimizes the objective function and creates soft boundaries between data points	Not capable of handling high dimensional data Can easily get stuck in the local minima, when finding the global minima	*Near O(N)*	Arbitrary
BIRCH	1. Multilevel clustering—for micro- and macro-level clustering to reduce complexity, and allow for enough flexibility respectively 2. Finds a good cluster with a single scan and improves continually 3. We can incrementally add new data points to the CF tree	Best clustering is achieved having more available memory and time constraints but capable of handling high dimensional data	*O(N)* (time)	Spherical

Abbreviations: BIRCH, Balanced Iterative and Clustering using Hierarchies; CF, clustering feature.

## Summary and Recommendations

**Figure 4.**
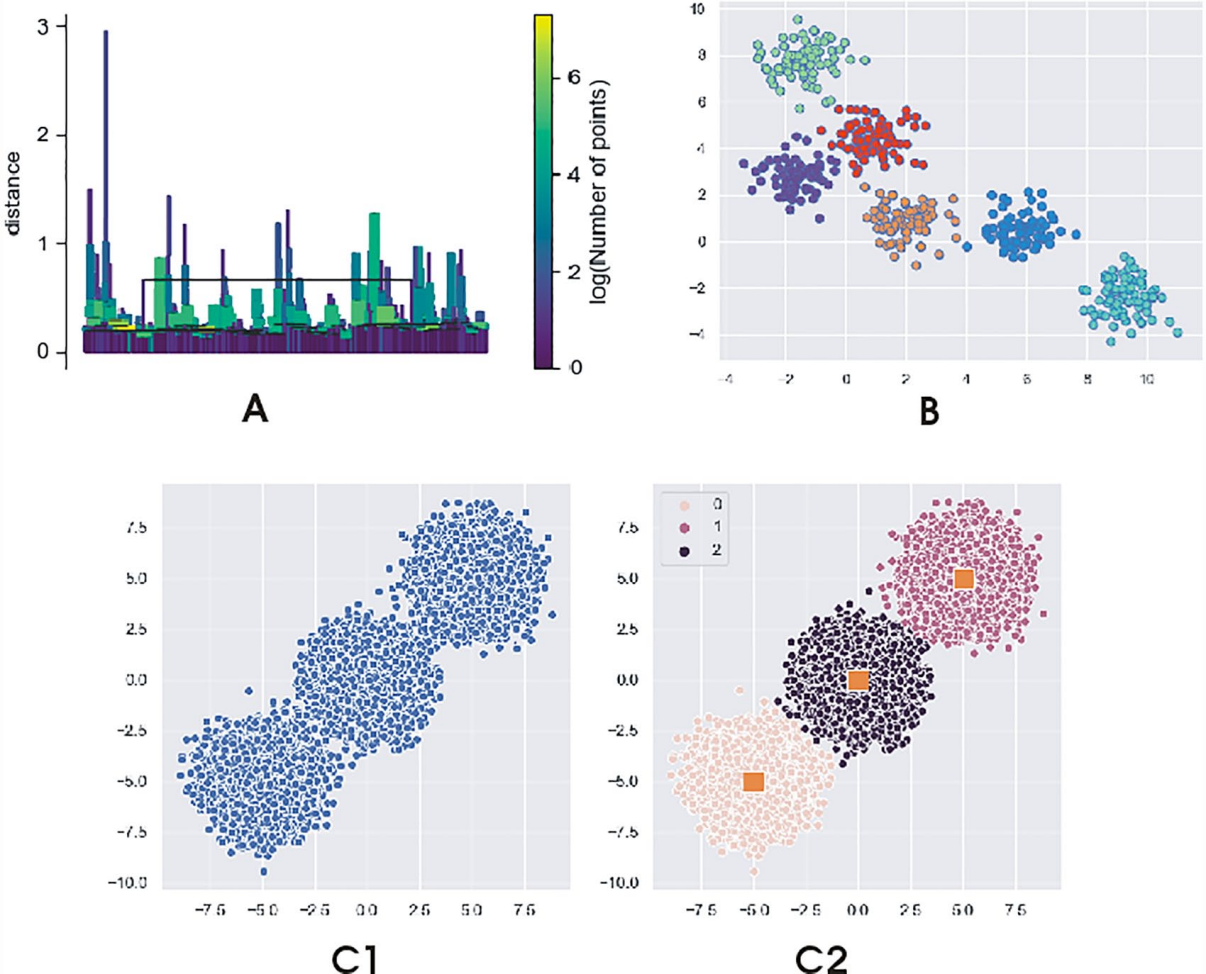
Clustering algorithm: Output from Python program showing (A) density-based algorithmic implementation with bars representing different densities; (B) BIRCH output showing clustering based on cluster radius and distance; (C) Fuzzy-C with C1 as the entry data and C2 showing membership association.^25,29^

Clustering is a useful bioinformatics algorithmic technique that has been applied in many areas of biology and medicine such as profiling the mycobacterium tuberculosis, detecting the size and stages of breast cancer, discovery of subtypes of parkinsonism, and distinguishing between asthma and normal cells from genome-wide transcriptional patterns.^1^ Denclue, Fuzzy-C, and BIRCH are examples of clustering algorithms that, although have different implementation and time complexity, can be used to provide solutions for different problems. From the implementation output in Figure 4, BIRCH generated clusters that were more spherically shaped, unlike Denclue and Fuzzy-C that have been suggested to generate arbitrary clusters (Table 1’).^27^ While Denclue and Fuzzy-C have trouble dealing with outliers, BIRCH has the best time complexity and the advantage of limiting input/output and progressively grouping multidimensional metric to produce the best subclusters which overall improves clustering quality. On the contrary, Fuzzy-C can handle overlapping data sets, and Denclue can handle data sets with a lot of noise because it allows for compact description of irregularly shaped clusters in a data set with high dimension, whereas BIRCH may not give a real-life simulation of data set. In recent literature, modified versions of these algorithms have been applied to cluster various data sets. A comparison between the three algorithm can be seen in Table 1. The multiple Fuzzy-C means have been applied to health data set for medical diagnoses of headache,^28^ BIRCH has been applied to cluster data sets of different time points,^24^ and Denclue algorithm (Denclue-IM) has been used in spam base data set to classify e-mail as spam or nonspam.^17^

Clustering analysis is limited in that there is no one clustering algorithm that works best for all solution. Also the use of traditional clustering algorithm with multilayer omics data which collect various types of omics information on the same subjects is challenging because while some clustering algorithms are good with text data, others are better with other types of data. The ideas from clustering could as well be useful in ongoing determination of different research subquestions. An interesting aspect is the transitioning from traditional clustering methods to computational techniques, and this could be used with respect to different data set. Future improvements to these algorithms should improve on their limitations to continuously broaden their applicability. In all, for every clustering problem, a more appropriate algorithm should be used.
